# Sir Henry Halford. Bart. G.C.H.

**Published:** 1845-01

**Authors:** 


### SIR HENRY HALFORD. BART. G.C.H.

SIR HENRY HALFORD was the son of Dr. James Vaughan of Leicester, and was born on the 2d of October, 1766. He received his preliminary education at Rugby, and graduated at Christ Church College, Oxford, in the year 1794. He established himself as physician in London, and quickly rose to great eminence in his profession. Distinguished no less by the elegance of his manners than by the extent of his classical acquirements, his practice rapidly increased, and he soon became the favorite physician of the Court, and of the elevated circles of rank and fashion. In 1809, he came into possession of an ample fortune on the death of Sir Charles Halford, a cousin of his mother, who was the daughter of Lord St. John; and in consequence of this acquisition, he changed his name from Vaughan to Halford, a baronetcy being at the same time conferred upon him by George the Third, who had previously appointed him his physician. He continued to hold a similar appointment under George the Fourth, William the Fourth, and her present Majesty; and thus enjoyed the remarkable distinction of having possessed the confidence of four successive sovereigns, and of having attended professionally almost every member of the Royal Family.

To the honours thus emanating from royal favour, were added the highest which the members of his own profession could confer. Sir Henry Halford was elected President of the College of Physicians in the year 1820; and held this office till his death, which happened in the month of March of the present year (1844). His constitution was naturally robust, and its vigour was sustained unimpaired to an advanced period of life; but in later years, the advance of age had become perceptible; his activity declined, and his constitution at last sunk, in the 78th year of his age, under the exhaustion consequent on a neuralgic affection.

He was elected Fellow of the Royal Society in 1810; he contributed no paper to our Transactions, but has enriched those of the College of Physicians with numerous valuable essays on professional subjects. His orations pronounced before the College are remarkable for the classical purity and elegance of their Latinity. (Proceedings of the Royal Society, 1844-5).

